# Opportunistic CT-Based Sarcopenia Screening: Masseter Muscle Index as a Prognostic Indicator of Prolonged Hospitalization in Carotid Endarterectomy

**DOI:** 10.3390/diagnostics16091378

**Published:** 2026-05-01

**Authors:** Sultan AlSheikh, Othman Alabdullah, Ghadah Alarify, Mohammed Ibn Saqyan, Kaisor Iqbal, Abdulmajeed Altoijry

**Affiliations:** 1 Division of Vascular Surgery, Department of Surgery, College of Medicine, King Saud University, Riyadh 11461, Saudi Arabia; sualsheikh@ksu.edu.sa (S.A.);; 2 College of Medicine, King Saud University, Riyadh 11461, Saudi Arabiamohamedsaqyan@gmail.com (M.I.S.)

**Keywords:** carotid endarterectomy, sarcopenia, frailty, masseter muscle index, hospital length of stay

## Abstract

**Background:** Carotid endarterectomy (CEA) plays a critical role in stroke prevention, but assessing a patient’s preoperative physiological reserve remains challenging. This study aimed to evaluate the impact of sarcopenia and preoperative albumin on postoperative management outcomes and resource utilization in CEA patients with a high prevalence of metabolic comorbidities. **Methods:** This retrospective cohort study evaluated 67 patients who underwent elective or urgent CEA between January 2015 and June 2025. Sarcopenia was quantified using the Masseter Muscle Index (MMI) derived from routine preoperative head and neck computed tomography (CT) scans. Multivariable regression models were used to assess the relationships between the MMI, serum albumin levels, and surgical outcomes. **Results:** The cohort had a mean age of 66.8 years and demonstrated a significant metabolic burden, with a high prevalence of diabetes (71.6%) and an average body mass index (BMI) of 28.15 kg/m^2^. Despite this predominantly overweight demographic, the MMI revealed underlying frailty and showed a strong inverse relationship with hospital resource utilization. A one-unit increase in the MMI significantly reduced total hospital length of stay (LOS) by 14.40 days (*p* = 0.001) and ICU LOS by 6.91 days (*p* < 0.001). Emergency surgery was the only independent predictor of mortality (OR 16.61, *p* = 0.047), while neither the MMI nor albumin significantly predicted short-term adverse clinical events. **Conclusions:** In a patient population where a higher BMI may mask underlying frailty, opportunistic screening for sarcopenia using routine preoperative CT scans provides important prognostic value. In this cohort study, a lower MMI showed an association with prolonged hospital and ICU stays; while it did not independently predict short-term mortality, its potential utility in forecasting resource utilization warrants further investigation in larger, prospective cohorts.

## 1. Introduction

Carotid endarterectomy (CEA) is the cornerstone intervention for stroke prevention, yet it is associated with a combined risk of 30-day stroke, myocardial infarction, and mortality rate of 3% to 6% [1,2]. These complications can have a substantial impact on long-term survival and functional outcomes [3]. Standard risk assessment tools using demographics and comorbidities may not adequately represent a patient’s ability to tolerate surgery or their risk of complications [4]. Therefore, there is a critical need for objective, prognostic biomarkers to assess preoperative frailty.

Sarcopenia, defined as reduced skeletal muscle mass, serves as an objective indicator of physiological reserve. In populations with high rates of metabolic comorbidities and elevated BMI, frailty is often inaccurately judged by standard clinical or visual assessment [5,6,7]. Therefore, the use of objective imaging methods is essential. Measuring muscle mass using routine preoperative CT scans is reliable, these scans are widely available, and the procedure allows for screening without any extra costs or exposure to radiation [8].

It has been found in several studies that sarcopenia is an independent risk factor for complications and mortality in surgery [6,9], including vascular procedures such as aortic repair and lower extremity revascularization [10,11]. Recent evidence indicates that sarcopenia can help predict outcomes after CEA. Reduced cranial muscle mass has been linked to prolonged hospitalization, complications, and recurrent stroke [12]. Similarly, lower cervical muscle density is independently associated with poorer overall survival, even after adjusting for age and ASA classification [13].

Nutritional status is another critical determinant of surgical outcomes, with serum albumin serving as a surrogate marker for nutrition and inflammation [14]. Hypoalbuminemia consistently predicts complications in general surgery [15] and adverse outcomes, including mortality and reintervention, in vascular surgery [16]. Specific to CEA, hypoalbuminemia has been identified as an independent predictor of perioperative stroke, myocardial infarction, and death [4]. Sarcopenia and hypoalbuminemia frequently coexist, potentially reflecting shared mechanisms of global frailty [17]. While evidence supports their individual prognostic significance, existing risk tools often lack these objective measures [18].

Given the routine availability of head and neck CT imaging prior to CEA, this study aimed to evaluate the diagnostic utility of the Masseter Muscle Index (MMI) as an opportunistic imaging biomarker for frailty. Specifically, we examined the independent and synergistic effects of the CT-derived MMI and preoperative serum albumin levels in predicting resource utilization, hospital length of stay, and postoperative outcomes in a cohort characterized by a substantial metabolic burden.

## 2. Materials and Methods

### 2.1. Study Design and Setting

This retrospective cohort study was conducted at King Khalid University Hospital (KKUH), a tertiary care center in Riyadh, Saudi Arabia. This study was conducted in accordance with the Strengthening the Reporting of Observational Studies in Epidemiology (STROBE) guidelines [19]. All procedures were performed in line with the Declaration of Helsinki and its later amendments. The study protocol was approved by the Institutional Review Board (No. E-23-7516), and the requirement for informed consent was waived due to the retrospective nature of the research and the use of anonymized data.

### 2.2. Study Population

All adult patients (≥18 years of age) who underwent elective or urgent CEA between 1 January 2015 and 30 June 2025 were screened for eligibility. Patients were included if they had a preoperative CT scan of the head and neck performed within six months prior to surgery, provided that the image quality was sufficient to allow for accurate masseter muscle measurement. Patients were excluded if their CT imaging was unavailable, incomplete, or if the image was of inadequate quality, or if they had a history of head and neck malignancy or prior major facial or mandibular surgery.

### 2.3. Data Collection and Diagnostic Measurements

Demographic and clinical variables such as age, sex, body mass index (BMI), comorbidities, smoking status, carotid stenosis characteristics, and operative details were extracted from electronic medical records. Additionally, preoperative serum albumin (g/L) was systematically collected from routine preoperative laboratory tests. Albumin was preselected as a well-established surrogate marker for baseline nutritional and inflammatory status to be evaluated alongside sarcopenia.

Sarcopenia was evaluated opportunistically using the Masseter Muscle Index (MMI), following the measurement protocol established by van Heusden et al. [20]. Preoperative axial CT images were analyzed using the institutional picture archiving and communication system (PACS). CT measurements were taken by a trained investigator who was blinded to the patients’ clinical data and postoperative outcomes. To evaluate intraobserver reliability, the primary investigator re-measured a random subset of scans after a minimum interval of two weeks, blinded to the initial measurements. To ensure interobserver reliability, a subset of scans was re-measured by a second independent, blinded investigator. Both intraobserver and interobserver reliability were assessed using the intraclass correlation coefficient (ICC) based on a two-way random-effects model with absolute agreement.

**Figure 1:**
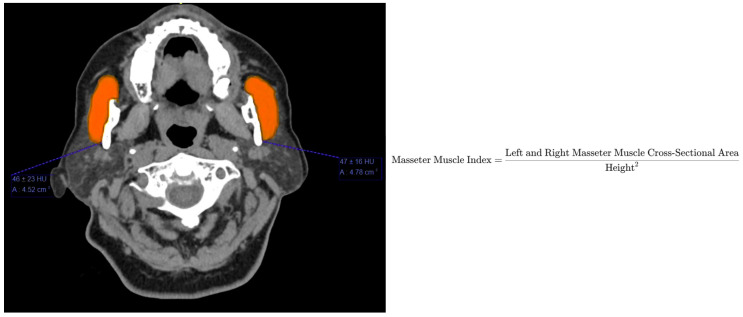
Axial computed tomography (CT) image demonstrating the measurement of the bilateral masseter muscle cross-sectional area (CSA). Measurements were taken at the anatomical level where the entire mandibular notch was visible. The orange-highlighted regions indicate the left and right masseter muscles.

The cross-sectional area (CSA) of the masseter muscles was measured bilaterally at the axial level where the entire mandibular notch was visible, ensuring a standardized and reproducible anatomical landmark. The mean CSA of the left and right muscles was calculated and normalized to the square of the patient’s height (cm^2^/m^2^) to derive the final MMI (Figure 1). Sarcopenia was subsequently defined using sex-specific MMI thresholds derived from standard deviation classifications of the study population, categorizing patients into Low (<mean − 1 SD), Average (mean ± 1 SD), and High (>mean + 1 SD) muscle mass groups.

### 2.4. Outcome Measures

To evaluate the prognostic utility of the MMI in predicting postoperative recovery and healthcare burden, the primary outcomes of this study were continuous markers of resource utilization: total hospital length of stay (LOS) and intensive care unit (ICU) LOS. Secondary outcomes included postoperative morbidity (defined as the occurrence of any major complication such as stroke, myocardial infarction, cardiac arrhythmia, surgical site infection, or reoperation), hospital readmission, and all-cause mortality.

### 2.5. Statistical Analysis

Descriptive statistics were used to summarize patient characteristics, sarcopenia indices, operative variables, and outcomes. Frequencies and percentages were reported for categorical variables, while means, standard deviations, medians, and ranges were used for continuous variables. Group comparisons for categorical variables were conducted using Chi-square tests or Fisher’s exact tests, as appropriate. Independent-sample t-tests or one-way ANOVA was utilized for normally distributed continuous variables, while Mann–Whitney U tests were applied to skewed distributions. Pearson’s correlation coefficients were calculated to examine the initial associations between muscle indices (MMI), preoperative albumin, and length-of-stay outcomes. There were no missing data for the primary variables of interest (MMI and albumin); thus, a complete-case analysis was utilized for the total cohort (*N* = 67). To evaluate the independent prognostic value of sarcopenia, multivariable linear regression models were constructed for the continuous primary outcomes (total hospital LOS and ICU LOS). Because LOS variables typically exhibit right-skewed distributions, the data were formally assessed prior to modeling. Diagnostic evaluations (Q-Q plots and scatterplots) confirmed that the assumptions of homoscedasticity and normality of the model residuals were adequately met, justifying the use of standard multivariable linear regression to provide directly interpretable clinical metrics (days). For the linear regression evaluating ICU length of stay, the analysis was explicitly restricted to the subset of patients who required ICU admission postoperatively (*n* = 27). These models utilized a forced-entry method, adjusting a priori for clinically relevant confounders (age and preoperative albumin). This variable selection strategy was strictly literature-driven rather than data-driven; age and albumin were systematically forced into the models alongside MMI due to their universally validated roles as foundational markers of baseline surgical frailty and risk [4,18]. To strictly prevent model overfitting given the small sample size, the number of independent variables per regression model was limited to a maximum of three predictors. Reductions in LOS are reported as unstandardized beta coefficients. For secondary binary outcomes (mortality, postoperative complications, readmission, and reintervention), multivariable binary logistic regression models were fitted, with adjustments for confounders including age, baseline comorbidities (hypertension, diabetes, prior stroke/TIA incidence), degree of carotid stenosis, emergency surgery status, preoperative albumin, and MMI. Additionally, to account for differences in baseline patient acuity and the inherent risks of urgent interventions, emergency surgery status was included as a predefined covariate in these multivariable models. To prevent model overfitting given the sample size, the number of independent variables per regression model was strictly limited. Multicollinearity among the predictors was assessed prior to modeling, with Variance Inflation Factors (VIFs) confirming acceptable limits. A *p*-value of less than 0.05 was considered statistically significant for all inferential analyses. All statistical analyses were performed using SPSS Statistics (version 30.0). A post hoc power analysis was conducted for the primary outcome utilizing the observed effect size (*f*^2^) derived from the multivariable linear regression coefficient of determination (*R*^2^), evaluating power given alpha = 0.05, the number of predictors, and the total sample size.

## 3. Results

### 3.1. Patient Characteristics and Operative Details

**Figure 2:**
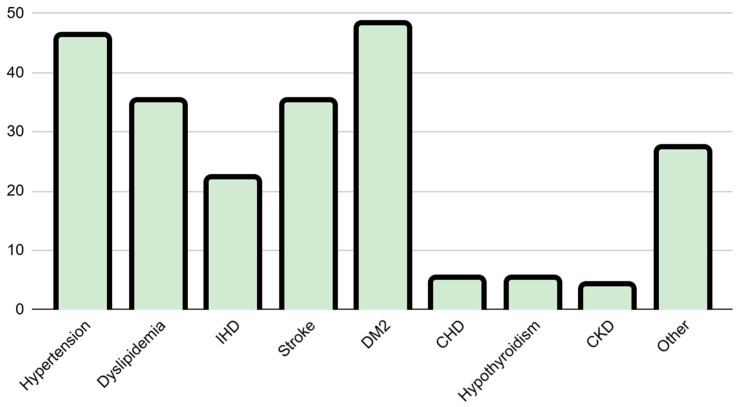
Medical comorbidities within the study cohort (*N* = 67).

This study included 67 patients who underwent CEA. The cohort had a mean age of 66.81 ± 10.32 years and a slight male predominance (55.2%), and the majority of patients were Saudi nationals (89.6%). The mean BMI of the cohort was 28.15 ± 5.09 kg/m^2^, classifying the average patient as overweight. Additionally, the cohort carried a substantial metabolic and comorbid burden: 71.6% of patients had diabetes, 68.7% had hypertension, and 52.2% had dyslipidemia. A history of stroke or transient ischemic attack (TIA) was present in 55.2% of the patients (Table 1, Figure 2). Regarding operative characteristics, the median degree of carotid stenosis was 90%, and the pathology was right sided in 56.7% of cases. Most procedures were elective (88.1%), while 11.9% were performed as emergency surgeries (Table 2).

### 3.2. Diagnostic Baseline: Sarcopenia and Albumin

**Figure 3:**
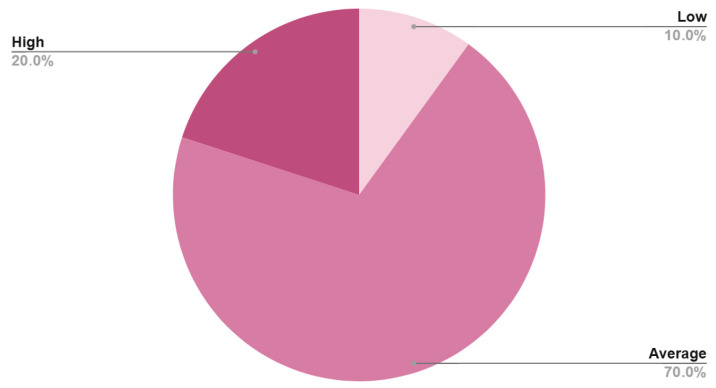
Distribution of Masseter Muscle Index (MMI) categories (Low, Average, and High) among female patients (*n* = 30).

**Figure 4:**
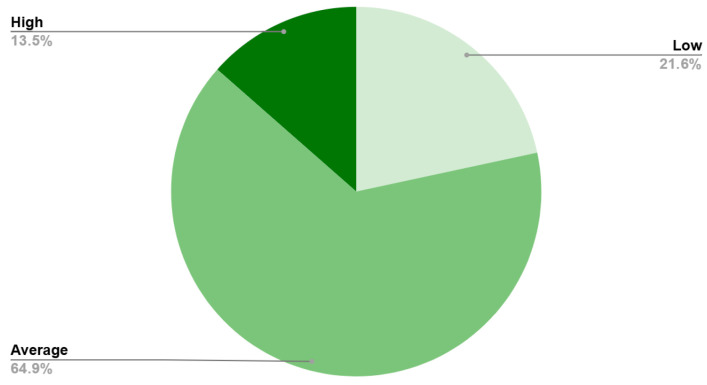
Distribution of Masseter Muscle Index (MMI) categories (Low, Average, and High) among male patients (*n* = 37).

The mean preoperative serum albumin level for the cohort was 33.87 ± 4.85 g/L (Table 3). Sarcopenia was evaluated opportunistically using the CT-derived MMI, which showed a mean score of 2.53 ± 0.60 for females and 2.86 ± 0.63 for males (Table 3). Based on standard deviation classifications, most patients were categorized as having “Average” muscle mass (70.0% of females and 64.9% of males), whereas 10.0% of females and 21.6% of males were classified in the “Low” muscle mass category (Table 3, Figure 3 and Figure 4). Both intraobserver (ICC = 0.95, 95% CI: 0.91–0.98) and interobserver agreement (ICC = 0.93, 95% CI: 0.88–0.96) were found to be excellent.

### 3.3. Primary Outcomes: Diagnostic Predictors of Resource Utilization

Overall, the mean total hospital length of stay (LOS) was 12.51 ± 23.50 days, and the mean ICU LOS was 4.74 ± 6.44 days. ICU admission was required for 40.3% of the cohort. Pearson’s correlation analysis demonstrated a significant inverse relationship between the MMI and continuous markers of resource utilization: total hospital LOS (r = −0.371, *p* = 0.002) and ICU LOS (r = −0.630, *p* < 0.001) (Table 4A). In the multivariable linear regression models, adjusted for age and preoperative albumin, the MMI emerged as a robust independent predictor of hospitalization duration (Table 5A). For every one-unit increase in the MMI, there was an associated reduction of 14.40 days in total hospital LOS (*p* = 0.001) and a reduction of 6.91 days in ICU LOS (*p* < 0.001). Subgroup analysis further revealed that patients in the “Low” MMI category had a significantly longer mean ICU stay (14.50 ± 11.73 days) compared with those in the “Average” (3.16 ± 3.44 days) or “High” (2.50 ± 1.29 days) categories (*p* = 0.002) (Table 5B). A similar trend was observed for total hospital LOS, where the “Low” group averaged 27.45 days, compared with 10.40 days for the “Average” group, which approached statistical significance (*p* = 0.058). A post hoc power analysis confirmed that given the sample size of 67, an alpha of 0.05, and the observed model *R*^2^ of 0.17, the study achieved a robust statistical power of 88% to detect the independent effect of the MMI on hospital length of stay.

### 3.4. Secondary Outcomes: Postoperative Complications and Mortality

The overall postoperative complication rate was 43.3%, and the all-cause mortality rate was 6.0%. The most frequent complications among those affected were hemorrhagic events (37.9%), followed by cerebrovascular accidents (20.7%) and acute kidney injury (13.8%). The hospital readmission rate was 46.3%, and 11.9% of patients required reintervention (Table 3). In the multivariable binary logistic regression models, the MMI was not a statistically significant diagnostic predictor for mortality (*p* = 0.100), postoperative complications (*p* = 0.497), readmission (*p* = 0.684), or reintervention (*p* = 0.876) (Table 6A). Similarly, preoperative albumin categories showed no significant associations with total hospital LOS (*p* = 0.285), ICU LOS (*p* = 0.491), postoperative complications (*p* = 0.105), or mortality (*p* = 1.00) (Table 4B). Emergency surgery was identified as the sole significant independent predictor of mortality in the broader multivariable analysis (OR 16.61, *p* = 0.047) (Table 6B).

## 4. Discussion

Although sarcopenia was not a significant predictor of perioperative mortality or complications, it was a reliable independent predictor of resource utilization. Specifically, a lower MMI was strongly associated with prolonged total hospital and ICU length of stay. Conversely, preoperative serum albumin levels did not demonstrate a significant association with postoperative outcomes or hospitalization duration in our study.

The most significant finding of our analysis was the inverse relationship between muscle mass and hospital duration. Patients with a low MMI required, on average, a significantly longer stay in the ICU compared with those with average muscle mass. This aligns with a growing body of evidence suggesting that sarcopenia is a marker of physiological frailty that impedes postoperative recovery [6]. In the context of CEA, our findings corroborate the work of Hogenbirk et al., who reported that reduced masseter muscle area was independently linked to prolonged hospitalization [12]. Sarcopenic patients often exhibit reduced metabolic reserve and higher vulnerability to surgical stress [7,9]. Consequently, preoperative identification of a low MMI could be clinically useful for planning a patient’s discharge and the number of hospital beds being utilized, allowing institutions to anticipate prolonged care needs for frail patients.

It is also important to clinically contextualize the relatively large effect size observed in our linear models (a 14.4-day reduction in total LOS per unit increase in MMI). This substantial magnitude likely reflects the disproportionate impact of severe frailty on recovery trajectories. The most severely sarcopenic patients frequently suffer compounding, cascading postoperative issues that result in extreme, outlier-driven hospital stays (as reflected by the wide standard deviation of 23.5 days in our total LOS). Therefore, while the exact day-count reduction should be interpreted cautiously as an average, the profound clinical signal remains clear: severe muscle depletion is inextricably linked to compounded healthcare burdens.

The prognostic value of opportunistic sarcopenia screening is increasingly supported by recent studies performed across a range of surgical disciplines. While traditional body composition analysis relies on lumbar imaging, recent cohort studies, such as Seğmen et al.’s study performed in 2025 [21], validate that cross-sectional masseter muscle parameters are reliable predictors of prolonged ICU and hospital stays, reinforcing the clinical utility of cranial CT scans when abdominal imaging is unavailable. Furthermore, our observation that sarcopenia primarily drives resource utilization rather than short-term mortality aligns with a recent extensive meta-analysis performed by Luo et al. [22], where they demonstrated that reduced muscle mass significantly increases the risk of adverse postoperative events and hospital burden in elderly surgical patients. This phenomenon is highly relevant to vascular interventions; as highlighted in a comprehensive review by Furukawa [23], sarcopenia is a critical determinant of physiological reserve, systemic vulnerability, and functional trajectories in vascular surgery, underscoring the necessity for objective frailty screening to optimize perioperative management.

In contrast to several large-scale observations in vascular surgery [10,11], we did not find a statistically significant association between sarcopenia and perioperative mortality or the composite endpoint of major complications in our study. This divergence may be attributable to sample size limitations; with a cohort of 67 patients and a relatively low mortality rate (6.0%), the study may have been underpowered to detect differences in binary clinical endpoints. Additionally, the specific definition of sarcopenia varies across the literature. While we utilized the MMI, a validated metric for head and neck imaging, other studies have relied on cervical muscle density. For instance, Bradley et al. noted that cervical muscle density, rather than area alone, was predictive of survival, suggesting that muscle quality (steatosis) might be as relevant as muscle quantity in this population [13].

We also found no significant correlation between preoperative albumin levels and postoperative outcomes. This contrasts with Stoner et al.’s findings, who identified hypoalbuminemia as a strong predictor of stroke and death after CEA in the NSQIP database [4]. Several factors may explain this discrepancy: First, the mean albumin level in our cohort was 33.87 ± 4.85 g/L, with a median of 34.7 g/L, suggesting a relatively uniform nutritional status with fewer patients in the severe hypoalbuminemia range compared with larger, multi-institutional datasets. Second, albumin is a negative acute-phase reactant with a long half-life; therefore, it may not acutely reflect the dynamic inflammatory state or rapid nutritional changes immediately before surgery as effectively as other markers might [14].

Our cohort had a high prevalence of metabolic comorbidities, where 71.6% of patients had diabetes and a mean BMI of 28.15 kg/m^2^, placing the average patient in the overweight category. The coexistence of high BMI and low muscle mass in our population highlights the importance of using CT-based body composition analysis rather than BMI alone, as BMI fails to distinguish between lean body mass and adipose tissue [8]. This clinical entity, defined by recent international consensus as sarcopenic obesity, is increasingly recognized as a significant hidden risk factor in surgical disciplines, because excess adiposity visually and clinically masks the severe depletion of skeletal muscle mass [5].

This study has several limitations that must be acknowledged. First, its retrospective, single-center design and relatively small sample size limit the generalizability of the findings. Furthermore, the inclusion of emergency cases (11.9%) introduces inherent clinical heterogeneity into the cohort. Urgent cases present with worse baseline acuity and follow distinct management pathways compared to elective cases. While emergency status was adjusted for in our broader regression models, the limited sample size precluded a statistically powered sensitivity analysis excluding these patients. Therefore, this heterogeneity remains a recognized limitation of our analysis. While major adverse events like stroke and mortality are the primary clinical endpoints of interest in carotid interventions, cohorts with very large numbers of patients are needed to detect statistically significant differences in these rare events. Consequently, the reliance on length of stay as a primary endpoint is a necessary limitation, as LOS is multifactorial and influenced by non-clinical, logistical factors. However, given the low incidence of mortality in this cohort (n = 4), LOS provided the most viable continuous measure to assess recovery burden. Furthermore, as confirmed by our power analysis, the sample size was significantly underpowered to detect independent predictors for rare secondary binary endpoints, such as short-term mortality. Second, our decision to exclude patients without suitable preoperative head/neck CT scans may have introduced selection bias. Third, while the MMI is a convenient tool for CEA patients, it measures a smaller muscle group compared with the psoas or Lumbar Skeletal Muscle Index.

## 5. Conclusions

In patients undergoing carotid endarterectomy, sarcopenia, defined by the Masseter Muscle Index, may serve as a potential prognostic indicator of prolonged ICU and hospital length of stay, acting as a surrogate for physiological reserve and recovery potential. In this exploratory cohort, a lower MMI was independently associated with prolonged hospital stays. While it did not independently predict short-term mortality or other management outcomes, its exploratory utility in forecasting resource utilization is valuable for preoperative planning. This study’s findings support further investigation into the integration of opportunistic sarcopenia screening using routine preoperative CT scans to identify frail patients who may benefit from prehabilitation or enhanced perioperative support.

## Figures and Tables

**Table 1:** Descriptive characteristics of the sample, *n* = 67.

Characteristics	Frequency (%) or Mean ± SD [Range]
**Demographics**	
Sex	
Female	30 (44.8%)
Male	37 (55.2%)
Age (at time of operation, years)	66.81 ± 10.32 [34–95]
Age category	
31–40	1 (1.5%)
41–50	2 (3.0%)
51–60	12 (17.9%)
61–70	33 (49.3%)
71–80	11 (16.4%)
>80	8 (11.9%)
Nationality	
Saudi	60 (89.6%)
Non-Saudi	7 (10.4%)
BMI (kg/m^2^)	28.15 ± 5.09 [16.84–43.18]
BMI category	
Underweight	2 (3.0%)
Normal weight	18 (26.9%)
Overweight	24 (35.8%)
Obesity Class I	17 (25.4%)
Obesity Class II	5 (7.5%)
Obesity Class III	1 (1.5%)
Smoking status	
Smoker	15 (22.4%)
Non-smoker	49 (73.1%)
Ex-smoker	3 (4.5%)

**Table 2:** Descriptive characteristics of the sample, *n* = 67.

Characteristics	Frequency (%) or Mean ± SD [Range]
Surgery	
American Society of Anesthesiologists (ASA)	
1	4 (6.0%)
2	10 (14.9%)
3	42 (62.7%)
4	11 (16.4%)
Emergency	8 (11.9%)
Elective	59 (88.1%)
Shunt	49 (73.1%)
Estimated blood loss (mL)	104.40 ± 73.06 [20–500], median = 100
Intraoperative complication	2 (3.0%)
Hospital length of stay (days)	12.51 ± 23.50 [2–132], median = 5
Intensive Care Unit (ICU)	
ICU admission	27 (40.3%)
Length of stay in ICU (days)	4.74 ± 6.44 [1–25], median = 2
Postoperative Outcomes	
Postoperative complication	29 (43.3%)
Hemorrhagic complications ^(1)^	11 (37.9%)
Cerebrovascular accident	6 (20.7%)
Acute kidney injury	4 (13.8%)
Acute decompensated heart failure	3 (10.3%)
Surgical site infection	3 (10.3%)
Pneumonia	2 (6.9%)
Readmission	31 (46.3%)
Reintervention	8 (11.9%)
Mortality	4 (6.0%)

Note: ^(1)^ bleeding or hematoma.

**Table 3:** Descriptive characteristics of the sample, *n* = 67.

Characteristics	Frequency (%) or Mean ± SD [Range]	
Carotid Stenosis		
Laterality (carotid side)		
Left	29 (43.3%)	
Right	38 (56.7%)	
Degree of carotid stenosis (% of stenosis)	84.78 ± 9.43 [50–100], median = 90	
Preoperative albumin (g/L)	33.87 ± 4.85 [22.0–44.6], median = 34.7	
Masseter Muscle Index (MMI)	Females (*n* = 30)	Males (*n* = 37)
MMI score	2.53 ± 0.60 [1.32–3.53], median = 2.54	2.86 ± 0.63 [1.87–4.82], median = 2.87
MMI category ^(1)^		
Low	3 (10.0%)	8 (21.6%)
Average	21 (70.0%)	24 (64.9%)
High	6 (20.0%)	5 (13.5%)

Note: ^(1)^ MMI categories are Low (less than mean − 1 SD), Average (mean − 1 SD to mean + 1 SD), and High (more than mean + 1 SD).

**Table 4:** (**A**) Analysis of Masseter Muscle Index (MMI). (**B**) Analysis of preoperative albumin categories.

(**A**)			
**Variables**	**MMI Value,** **Mean ± SD**		**Statistical Test Results**
Mortality			t(65) = 1.88, *p* = 0.065
No	2.75 ± 0.63		
Yes	2.14 ± 0.27		
Total hospital length of stay	Correlation r = −0.371		*p* = 0.002
ICU admission			t(65) = 0.04, *p* = 0.965
No	2.71 ± 0.66		
Yes	2.72 ± 0.61		
Length of stay in ICU (days)	Correlation r = −0.630		*p* < 0.001
Postop. complications			t(65) = 0.77, *p* = 0.444
No	2.66 ± 0.64		
Yes	2.78 ± 0.63		
Readmission			t(65) = 0.38, *p* = 0.707
No	2.68 ± 0.71		
Yes	2.74 ± 0.55		
Reintervention			t(65) = 0.20, *p* = 0.841
No	2.71 ± 0.64		
Yes	2.75 ± 0.63		
Emergency			t(65) = 1.19, *p* = 0.237
No	2.75 ± 0.63		
Yes	2.46 ± 0.61		
(**B**)			
	**Preoperative Albumin Category ^1^**		**T-Test or Chi-Square/Fisher’s Exact Test Results**
	**Low** **(*n* = 33)**	**Normal** **(*n* = 34)**	
**Total hospital length of stay (days)**	9.39 ± 10.79	15.53 ± 31.18	t(65) = 1.08, *p* = 0.285
Length of stay in ICU (days)	3.60 ± 3.47	5.41 ± 7.71	t(25) = 0.70, *p* = 0.491
Mortality			Fisher’s exact test *p* = 1.00
No	31 (93.9%)	32 (94.1%)	
Yes	2 (6.1%)	2 (5.9%)	
Postop. complications			χ^2^(1) = 2.62, *p* = 0.105
No	22 (66.7%)	16 (47.1%)	
Yes	11 (33.3%)	18 (52.9%)	
Readmission			χ^2^(1) = 0.13, *p* = 0.720
No	17 (51.5%)	19 (55.9%)	
Yes	16 (48.5%)	15 (44.1%)	
Reintervention			Fisher’s exact test *p* = 1.00
No	29 (87.9%)	30 (88.2%)	
Yes	4 (12.1%)	4 (11.8%)	

**Note**: Reported values are mean ± SD or frequency (column %). ^1^ **Normal** albumin levels are in the range of 39–49 g/L.

**Table 5:** (**A**) Linear regression models with MMI. (**B**) Analysis of Masseter Muscle Index (MMI) categories.

(**A**)				
	**Model 7** **Outcome = Hospital Length of Stay (Days)**			**Model 8** **Outcome = Length of Stay in ICU**
Age	−0.07 (0.27), *p* = 0.789			−0.15 (0.13), *p* = 0.256
Preoperative albumin (g/L)	0.83 (0.58), *p* = 0.154			−0.01 (0.22), *p* = 0.956
Masseter Muscle Index (MMI)	−14.40 *** (4.27), *p* = 0.001			−6.91 *** (1.69), *p* < 0.001
Overall model fit	F(3,63) = 4.34, *p* = 0.008 R^2^ = 0.17			F(3,23) = 5.88, *p* = 0.004 R^2^ = 0.43
(**B**)				
	**Masseter Muscle Index (MMI) Category**			**ANOVA or Chi-Square/Fisher’s Exact Test Results**
	**Low/Very Low** **(*n* = 11)**	**Average** **(*n* = 45)**	**High/Very High** **(*n* = 11)**	
Total hospital length of stay (days)	27.45 ± 39.44	10.40 ± 19.96	6.18 ± 5.00	F(2,64) = 2.97, *p* = 0.058
Length of stay in ICU (days)	14.50 ± 11.73	3.16 ± 3.44	2.50 ± 1.29	F(2,24) = 8.54, *p* = 0.002
Mortality				Fisher’s exact test *p* = 0.242
No	9 (81.8%)	43 (95.6%)	11 (100%)	
Yes	2 (18.2%)	2 (4.4%)	0 (0%)	
Postop. complications				Fisher’s exact test *p* = 0.759
No	7 (63.6%)	26 (57.8%)	5 (45.5%)	
Yes	4 (36.4%)	19 (42.2%)	6 (54.5%)	
Readmission				χ^2^(2) = 1.02, *p* = 0.363
No	8 (72.7%)	23 (51.1%)	5 (45.5%)	
Yes	3 (27.3%)	22 (48.9%)	6 (54.5%)	
Reintervention				Fisher’s exact test *p* = 0.849
No	10 (90.9%)	40 (88.9%)	9 (81.8%)	
Yes	1 (9.1%)	5 (11.1%)	2 (18.2%)	

Note: Reported values are regression coefficient B (standard error); significance *** *p* < 0.001; Reported values are mean ± SD or frequency (column %).

**Table 6:** (**A**) Binary logistic regression models with MMI. (**B**) Binary logistic regression models.

(**A**)				
	**Model 1** **Outcome = Mortality**	**Model 2** **Outcome = Postop. Complications**	**Model 3** **Outcome = Readmission**	**Model 4** **Outcome = Reintervention**
Age	1.04 (0.91–1.18), *p* = 0.551	1.02 (0.97–1.07), *p* = 0.483	0.99 (0.94–1.04), *p* = 0.590	1.02 (0.94–1.10), *p* = 0.626
Preoperative albumin (g/L)	0.91 (0.73–1.14), *p* = 0.406	1.06 (0.95–1.18), *p* = 0.285	0.98 (0.89–1.09), *p* = 0.746	1.05 (0.89–1.24), *p* = 0.589
Masseter Muscle Index (MMI)	0.14 (0.01–1.45), *p* = 0.100	1.31 (0.60–2.85), *p* = 0.497	1.17 (0.54–2.53), *p* = 0.684	1.10 (0.34–3.50), *p* = 0.876
Overall model fit	χ^2^(3) = 5.27, *p* = 0.153 Nagelkerke R^2^ = 0.21	χ^2^(3) = 1.97, *p* = 0.578 Nagelkerke R^2^ = 0.04	χ^2^(3) = 0.48, *p* = 0.924 Nagelkerke R^2^ = 0.01	χ^2^(3) = 0.46, *p* = 0.927 Nagelkerke R^2^ = 0.01
(**B**)				
	**Model 5** **Outcome = Mortality**		**Model 6** **Outcome = Postop. Complications**	
Age	1.04 (0.93–1.17), *p* = 0.475		1.02 (0.96–1.07), *p* = 0.558	
Comorbidities				
Hypertension	0.60 (0.04–9.66), *p* = 0.721		0.42 (0.13–1.34), *p* = 0.144	
Diabetes	1.58 (0.09–28.05), *p* = 0.755		2.74 (0.78–9.64), *p* = 0.115	
Stroke	1.38 (0.10–18.42), *p* = 0.806		0.73 (0.25–2.15), *p* = 0.573	
Emergency				
No	Reference		Reference	
Yes	16.61 * (1.03–267.35), *p* = 0.047		5.48 (0.84–35.64), *p* = 0.075	
Degree of carotid stenosis (% of stenosis)	0.98 (0.87–1.11), *p* = 0.784		0.97 (0.92–1.03), *p* = 0.394	
Overall model fit	χ^2^(7) = 5.98, *p* = 0.542 Nagelkerke R^2^ = 0.24		χ^2^(7) = 9.97, *p* = 0.190 Nagelkerke R^2^ = 0.19	

Note: Reported values are odds ratios (95% CI); significance * *p* < 0.05.

## Data Availability

All data are available on request from the corresponding author.

## Abbreviations

The following abbreviations are used in this manuscript:

MMI	Masseter Muscle Index
CEA	Carotid Endarterectomy
LOS	Length of Stay
ICU	Intensive Care Unit
CT	Computed Tomography
